# Allogeneic chondrogenically differentiated human bone marrow stromal cells do not induce dendritic cell maturation

**DOI:** 10.1002/term.2682

**Published:** 2018-06-20

**Authors:** C.H. Kiernan, A. KleinJan, M. Peeters, E.B. Wolvius, E. Farrell, P.A.J. Brama

**Affiliations:** ^1^ Department of Oral and Maxillofacial Surgery, Orthodontics and Special Dental Care, Erasmus MC University Medical Center Rotterdam Rotterdam The Netherlands; ^2^ Department of Pulmonary Medicine, Erasmus MC University Medical Center Rotterdam Rotterdam The Netherlands; ^3^ School Of Veterinary Medicine, Veterinary Science Centre University College Dublin Dublin Ireland

**Keywords:** allogeneic, chondrogenesis, dendritic cell, endochondral ossification, immunogenicity, mesenchymal stem cell

## Abstract

Bone marrow stromal cell (BMSC)‐mediated endochondral bone formation may be a promising alternative to the current gold standards of autologous bone transplantation, in the development of novel methods for bone repair. Implantation of chondrogenically differentiated BMSCs leads to bone formation in vivo via endochondral ossification. The success of this bone formation in an allogeneic system depends upon the interaction between the implanted constructs and the host immune system. The current study investigated the effect of chondrogenically differentiated human bone marrow stromal cell (hBMSC) pellets on the maturation and function of dendritic cells (DCs) by directly coculturing bone forming chondrogenic hBMSC pellets and immature or lipopolysaccharide (LPS)‐matured DCs in vitro. Allogeneic chondrogenic hBMSC pellets did not affect the expression of CD80, CD86, or HLADR on immature or LPS‐matured DCs following 24, 48, or 72 hr of coculture. Furthermore, they did not induce or inhibit antigen uptake or migration of the DCs over time. IL‐6 was secreted by allogeneic chondrogenic hBMSC pellets in response to LPS‐matured DCs. Overall, this study has demonstrated that maturation of immature DCs was not influenced by allogeneic chondrogenic hBMSC pellets. This suggests that allogeneic chondrogenic hBMSC pellets do not stimulate immunogenic responses from DCs in vitro and are not expected to indirectly activate T cells via DCs. For this reason, allogeneic chondrogenic bone marrow stromal cell pellets are promising candidates for future tissue engineering strategies utilising allogeneic cells for bone repair.

## 1 INTRODUCTION

Bone marrow stromal cells (BMSCs) are multipotent stromal cells with the capacity to differentiate along adipogenic, osteogenic, and chondrogenic lineages (Pittenger et al., 1999). Our group and others have shown that implantation of chondrogenically differentiated BMSC pellets into immunodeficient and immunocompetent animals leads to bone formation in vivo via the process of endochondral ossification (Farrell et al., 2011; Gawlitta et al., 2010; Huang, Durbhakula, Angele, Johnstone, & Yoo, 2006; Scotti et al., 2013; Thompson, Matsiko, Farrell, Kelly, & O'Brien, 2014). Undifferentiated BMSCs have been shown to have unique immunomodulatory characteristics (Bernardo & Fibbe, 2013). BMSCs have low expression levels of MHC Class I and lack expression of MHC Class II and T cell costimulatory molecules CD80 (B7.1) and CD86 (B7.2) on their surface and thus have been shown to not stimulate allogeneic lymphocyte activation (Griffin et al., 2013; Le Blanc, Tammik, Rosendahl, Zetterberg, & Ringden, 2003; Lohan et al., 2014; Nauta et al., 2006; Ryan et al., 2014; Sudres et al., 2006). It is unclear whether these immunomodulatory properties are maintained upon differentiation. Allogenic chondrogenically differentiated BMSCs could be used in a therapy as an alternative to the current gold standard for bone repair, autologous bone transplantation. For this to become a feasible option, the interaction between the implanted cells and the host immune system in the process of the bone formation needs to be fully elucidated.

Although there have been many studies investigating the effects of undifferentiated BMSCs on host immune cells, little is known about the effect of chondrogenically differentiated BMSC pellets. Undifferentiated BMSCs are known to have an immunosuppressive effect on T lymphocytes (Di Nicola et al., 2002; Krampera et al., 2003; Mougiakakos et al., 2011). In a recent study by our group, direct coculture of chondrogenically differentiated BMSC pellets and peripheral blood mononuclear cells (PBMCs) revealed that chondrogenically differentiated BMSC pellets did not induce the proliferation of naïve or stimulated T lymphocytes, suggesting that allogeneic chondrogenically differentiated BMSC pellets are non‐immunogenic (Kiernan et al., 2016). However, it is possible that there might be an indirect stimulation of T cells by dendritic cells (DCs) that have encountered these BMSCs. DCs are the main antigen‐presenting cells (APCs) of the innate immune system, with a primary role in activating T‐cell‐dependent immune responses (Guermonprez, Valladeau, Zitvogel, Thery, & Amigorena, 2002). Therefore, the aim of the current research was to determine if chondrogenically differentiated BMSC pellets affect DCs.

Immature DCs survey the peripheral tissues for potential threats to the immune system, have low levels of major histocompatibility complex (MHC) molecules (or human leukocyte antigen [HLA]) and the costimulatory molecules CD80 and CD86, and are not yet equipped to stimulate naïve T cells. Antigen uptake and processing by immature DCs, in addition to locally produced inflammatory cytokines, induce their maturation and migration to the draining lymph nodes. DC maturation is a prerequisite for the stimulation of naïve and memory T cells (Lutz & Schuler, 2002). Therefore, DCs present themselves as potential targets for BMSC‐mediated immunosuppression. Undifferentiated BMSCs have been shown to inhibit the differentiation, maturation, and function of DCs (Jiang et al., 2005; Spaggiari, Abdelrazik, Becchetti, & Moretta, 2009; Zhang et al., 2004), however, little is known about the effect of chondrogenically differentiated BMSCs on DCs. Chondrogenically differentiated BMSCs have been reported to be more immunogenic compared with undifferentiated BMSCs (Chen et al., 2007; Ryan et al., 2014). However, few studies have directly cocultured chondrogenically differentiated BMSC pellets and immune cells. Undifferentiated BMSCs lack immunogenicity due to the absence of MHC Class I or costimulatory molecule expression on their surface, and their ability to secrete immunosuppressive molecules (PGE2, NO and IDO; Chen, Tredget, Wu, & Wu, 2008; Meisel et al., 2004; Ryan, Barry, Murphy, & Mahon, 2007; Sato et al., 2007). Chondrogenically differentiated BMSCs in contrast may lose this immunosuppressive capacity by upregulating costimulatory molecules on the surface, leading to induced DC maturation and enhanced lymphocyte proliferation (Chen et al., 2007). In addition to this, the anti‐inflammatory mediators PGE2 and NO have been shown to be significantly reduced upon chondrogenic differentiation of BMSCs, inducing immunogenicity in these cells (Ryan et al., 2014). Contradictory to these reports, Zheng, Li, Ding, Jia, and Zhu (2008) demonstrated that chondrogenically differentiated BMSCs maintain their immunosuppressive behaviour, to conclude that both undifferentiated and chondrogenically differentiated BMSCs suppressed T cell responses. Neither of these studies directly cocultured chondrogenic BMSC pellets with DCs. Therefore, it is clear that more research is required to confirm the immunomodulatory capacity of these cells to make them clinically applicable for bone tissue engineering. The aim of the current study was to analyse the effect of allogeneic chondrogenically differentiated human BMSC pellets on immature and lipopolysaccharide (LPS)‐matured DCs by directly coculturing them with allogeneic chondrogenically differentiated hBMSC pellets in vitro.

## 2 MATERIALS AND METHODS

### 2.1 Isolation, expansion, and chondrogenic differentiation of human bone marrow mesenchymal cells

Human bone marrow stromal cells (hBMSCs) were isolated as previously described (Kiernan et al., 2016) from heparinised femoral‐shaft marrow aspirates of patients undergoing total hip arthroplasty (with informed consent after approval by Erasmus MC medical ethical committee protocol METC‐2004‐142). hBMSCs were expanded in standard medium consisting of minimum essential medium alpha medium (Invitrogen, Carlsbad, CA, USA) supplemented with 10% heat‐inactivated fetal calf serum (Life Technologies; The Netherlands; lot number, 41Q2047K); 50‐μg/ml gentamycin; 1.5‐μg/ml fungizone (All Invitrogen); 1‐ng/ml fibroblast growth factor‐2 (Instruchemie B.V., Delfzijl, The Netherlands); and 0.1 mM of _L_‐ascorbic acid 2‐phosphate (Sigma‐Aldrich, MO, USA). hBMSCs were cultured at 37 °C under humidified condition and 5% carbon dioxide, refreshing medium every 3–4 days. Third to fourth passage cells were trypsinised using 0.05% trypsin (Life Technologies) when they reached 80–90% confluency and used for BMSC pellet cultures. hBMSCs (0.2 × 10^6^) were added to 15‐ml polypropylene tubes (Sarstedt) in 0.5 ml of standard chondrogenic medium consisting of high‐glucose Dulbecco's modified Eagle's medium with 50‐mg/ml gentamicin; 1.5‐mg/ml Fungizone (Invitrogen); 100‐mM sodium pyruvate (Sigma‐Aldrich); 1:100 ITS (BD Biosciences); _L_‐ascorbic acid 2‐phosphate; 10‐ng/ml TGF‐β3 (Peprotech); and 100‐nM dexamethasone (Sigma‐Aldrich). Cells were cultured for 10 days with TGFβ3, refreshing the medium every 3–4 days.

### 2.2 Generation and culture of monocyte‐derived DCs

PBMCs were isolated from buffy coats of healthy male blood donors (Sanquin Bloedvoorziening, Rotterdam, The Netherlands), using previously described methods (Kiernan et al., 2016). Briefly, peripheral blood was diluted 1:1 in wash medium (RPMI‐1640 medium containing 1.5‐μg/ml fungizone and 50‐μg/ml gentamycin) and divided into 50‐ml‐filtered falcon tubes containing Ficoll‐Paque PLUS (density 1.077 g/ml; GE Healthcare, Uppsala, Sweden) and centrifuged (690*g* no brake, 20 min). The plasma was carefully removed and discarded. The remaining layers above the filter were transferred to a new 50‐ml falcon tube and centrifuged at 835*g* for 7 min with brake. Following four washing steps (in wash medium, 690× *g* for 7 min), monocytes were separated from freshly obtained PBMCs using the MACS Monocyte Isolation kit (Miltenyi, Biotech) according to manufacturer's instructions. Briefly, 500 × 10^6^ PBMCs were incubated with 100‐μl CD14+ MicroBeads for 20 min in the dark at 4 °C, washed and resuspended in running buffer (phosphate buffered saline (PBS) with 0.5% BSA, Sigma, and 2‐mM EDTA, Gibco). Subsequently, magnetically labelled cells were applied on a column (LS column, Miltenyi, Biotech), pre‐washed with 5 ml of running buffer) and placed in a MidiMACS Separator (Miltenyi, Biotech). The CD14+ fraction was collected after removal of the column from the MidiMACS Separator while collecting the flow‐through. Afterwards, CD14+ monocytes were washed with RPMI‐1640 medium (supplemented with 50‐μg/mL Gentamycin and 1.5‐μg/mL Fungizone, both Invitrogen) and cultured in flat‐bottomed six‐well tissue culture plates at a concentration of 4 × 10^6^ cells in 3‐ml RPMI‐1640 medium supplemented with 10% FCS (heat‐inactivated, Gibco), 2‐mM L‐glutamine, 50‐μg/ml Gentamycin, 1.5‐μg/ml Fungizone (all Invitrogen), 50‐ng/ml GM‐CSF (Peprotech), and 10‐ng/mL IL‐4 (Peprotech) to induce DC differentiation. Every 2 days, half of the medium was refreshed, and monocyte‐derived DCs were cultured for 6 days (immature DCs). For induction of DC maturation, LPS (Sigma) was added at 100 ng/ml on Day 5 for 24 hr (LPS‐matured DCs).

### 2.3 Chondrogenically differentiated hBMSC coculture with immature and LPS‐matured DCs

Chondrogenic hBMSC pellets (0.2 × 10^6^ cells at the time of pellet formation) differentiated for 10 days were added to DCs cultured alone for 6 days (immature DCs), or alone for 5 days, and then stimulated with LPS for 24 hr (LPS‐matured DCs; 1 × 10^6^ cells) for 24, 48, and 72 hr in 24‐well plates containing supplemented RPMI‐1640.

### 2.4 Phenotypic analysis of DC populations using flow cytometry

Following above‐described coculture regimes, DCs were harvested by pipette aspiration. The chondrogenic hBMSC pellets were removed prior to the DC harvest. DCs were centrifuged for 8 min at 248*g*. The cells were resuspended in FACSflow (BD Biosciences), and 2 × 10^5^ cells per sample were transferred to FACS tubes. Cells centrifuged for 5 min at 689*g* were resuspended in 100 μl of FACSflow containing anti‐CD11c (clone B‐ly6; allophycocyanin), anti‐HLA‐DR (clone G46‐6; peridinin chlorophyll protein), anti‐CD86 (clone 2331; phycoerythrin), anti‐CD80 (clone L307.4; phycoerythrin‐Cy7), anti‐CD14 (fluorescein isothiocyanate [FITC]) antibodies (all BD Biosciences), and live and dead cell marker (Life Technologies; APC‐Cy7) and incubated for 30 min at 4 °C in the dark. Samples were washed twice with FACSflow (centrifuged for 5 min at 689*g*) and resuspended in 200 μl of FACSflow. Samples were analysed on a FACSJazz (BD Biosciences) and FlowJo software version 10.0.7 (Treestar Inc.).

### 2.5 Analysis of DC phagocytic uptake

To study the effect of chondrogenically differentiated hBMSC pellets on DC phagocytic uptake capacity, 5 × 10^5^ DCs were harvested and resuspended in 1 ml of PBS containing FITC‐Dextran (1 mg/ml; Sigma). Cells were transferred back to the 24‐well plates and incubated for 1 hr at 37 °C, or at 4 °C as a control. The DCs were then harvested; centrifuged (248*g*, 8 mins); and resuspended in ice‐cold PBS. Samples were washed twice with ice‐cold PBS and resuspended in 500 μl of FACSflow. Quantitative uptake of FITC‐Dextran was analysed by flow cytometry as above.

### 2.6 Analysis of DC migratory capacity

Cell migration was performed using 24‐well Transwell chambers (Corning Costar, Cambridge, MA) using 5‐μm pore size polycarbonate membranes. Immature and LPS‐matured DCs (1 × 10^5^ in 100 μl of supplemented RPMI‐1640 medium) cultured with chondrogenic hBMSC pellets were seeded into the upper chamber. Supplemented RPMI‐1640 medium (600 μl per well) with and without CCL21 (250 ng/ml) was added to the lower chamber. Cells were incubated for 3 hr at 37 °C. The number of migrated cells was quantified using Accucheck counting beads according to the manufacturer's instructions (Invitrogen). Briefly, cells were resuspended in 100 μl of FACSflow and an equal volume of Accucheck beads. The amount of counting beads was determined. Because the amount of counting beads in each samples is equal, the number of migrated cells was calculated. The number of DCs that migrated to normal medium was subtracted from those that migrated to the medium containing CCL21.

### 2.7 IL‐6, IL‐10, and IL‐12 secretion from supernatants from coculture

Supernatants from the cocultures were centrifuged at 690*g* for 10 min and stored at −80 °C for later analysis. IL‐6 (Peprotech), IL‐10 (R&D Systems), and IL‐12 (Peprotech) secretion was determined in the supernatants from the cocultures using enzyme‐linked immunosorbent assay measurements. The measurements were performed and calculated according to manufacturer's instructions.

### 2.8 Histological analysis of chondrogenic hBMSC pellets cultured with DCs

Chondrogenic hBMSC pellets cultured with and without DCs were harvested, washed in PBS, and then fixed in 4% formalin for 1 hr at room temperature. Following fixation, pellets were embedded in 3% agarose, processed, and embedded in paraffin. Sectioned slides were deparaffinised through alcohol series (xyleen, 100% ethanol, 96% ethanol, and 70% ethanol) and rinsed twice in distilled water. For thionine staining, sections were stained for 5 min with 0.04% thoinin in 0.01‐M aqueous sodium acetate (pH 4.5) followed by differential staining in 70% ethanol (+/−10 s), 96% ethanol (+/− 30 s), and 100% ethanol (1 min). For CD11c staining, antigen retrieval was first performed by heating samples to 80–90°C for 20 min in Dako heat antigen retrieval solution (S1699, Dako, Heverlee, Belgium). Nonspecific antibody binding was blocked using 1% milk block in 1% BSA in PBS solution. Sectioned slides were stained using a rabbit monoclonal anti‐CD11 (EP1347Y, Genetex) or rabbit IgG as a negative control antibody (X0903, Dako Cytomation) and labelled using an alkaline phosphatase link and label (Biogenex) to identify the presence of DCs within the matrix of the cells.

### 2.9 Quantitative real‐time reverse transcription polymerase chain reaction

Immature and LPS‐matured DCs were removed from the coculture, washed with PBS, and resuspended in TRIzol reagent (Thermo Scientific). Similarly, chondrogenically differentiated hMSCs were removed from the coculture, washed with PBS, and crushed in TRIzol reagent. RNA was isolated from all samples using RNeasy mini kit (Qiagen). Complementary DNA was synthesised from isolated RNA using first‐strand complementary DNA synthesis kit (Thermo Scientific) and used for real‐time reverse transcription polymerase chain reaction (PCR). Quantitative gene expression was determined using qPCR Mastermix Plus for SYBR Green I—dTTP (Eurogentec) for the genes IL‐6 (FW:TCGAGCCCACCGGGAACGAA and RV:GCAGGGAGGGCAGCAGGCAA) and CCR7 (FW:CAGCCTCCTGTGTGGTTTTAC and RV:CCAGCACGCTTTTCATTGGTT). Data are represented relative to the housekeeping gene GAPDH (the 2^‐∆CT^ method).

### 2.10 Statistics

Statistical analysis was performed using IBM SPSS Version 21 using a linear mixed model with Bonferroni post‐test, or GraphPad Prism v.5 for a paired *t* test or an unpaired *t* test as indicated in figures. Values are presented as mean ± standard deviation where *p* < .05 was considered statistically significant.

## 3 RESULTS

### 3.1 Chondrogenically differentiated hBMSC pellets do not induce DC maturation following 24 hr of coculture

**Figure 1:**
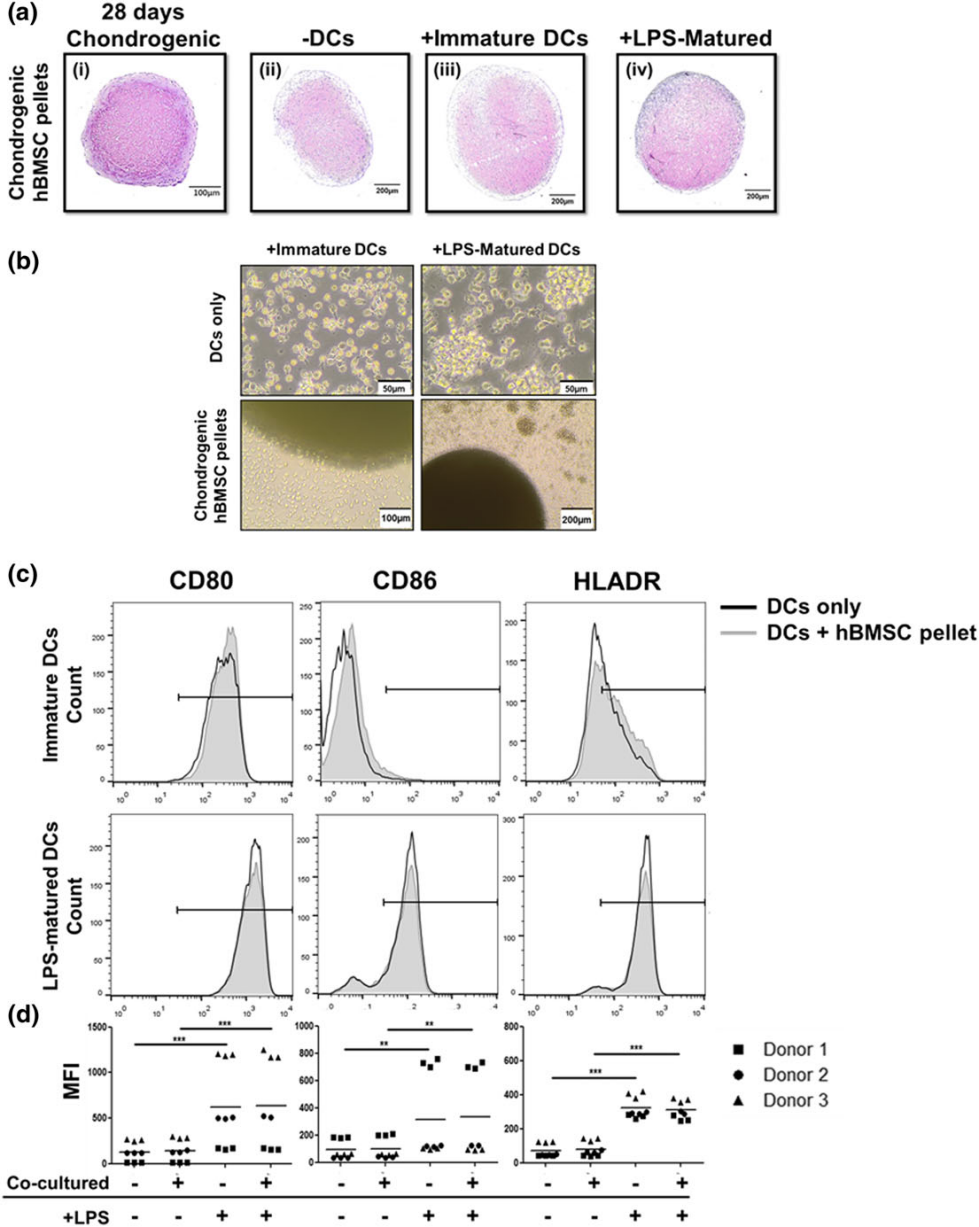
Chondrogenically differentiated human bone marrow stromal cell (hBMSC) pellets do not induce dendritic cell (DC) maturation following 24 hr in coculture. Immature or lipopolysaccharide (LPS)‐matured DCs were cultured alone (black) or with chondrogenically differentiated hBMSC pellets (grey) for 24 hr. (a [i]) Thionine staining confirmed the chondrogenicity of hBMSC pellets following 28 days in chondrogenic medium. (a [ii]) Following 24 hr in RPMI‐medium, hBMSC pellets were still chondrogenic. (a [iii] and [iv]) Following 24 hr of coculture with immature and LPS‐mature DCs, hBMSC pellets were still chondrogenic. (b) Microscopic images following 24 hr of culture demonstrate the morphology of the cocultured cells. (c and d) Flow cytometric analysis revealed no difference in the expression of the maturation markers CD80, CD86, and HLADR in immature DCs cocultured with chondrogenically differentiated hBMSC pellets. (c) Horizontal bars represent positive regions gated from unstained controls. (d) Data represented as the mean fluorescence intensity (MFI). *n* = 9 (three different hBMSC and DC donors in triplicate). Linear mixed model with Bonferroni post‐test ^*^ *p* < .05, ^**^ *p* < .005, and ^***^ *p* < .001 [Colour figure can be viewed at http://wileyonlinelibrary.com]

Chondrogenically differentiated hBMSCs cultured in 3D for 10 days were added to immature or LPS‐matured DCs for 24 hr (Figure 1a) at a ratio of one BMSC to five DCs. Thionine staining performed on hBMSC pellets confirmed that they were chondrogenic (Figure S1). Chondrogenically differentiated hBMSC pellets did not affect the expression of CD80, CD86, or HLADR in immature DCs (Figure 1b,c). DCs were successfully matured with the addition of LPS as shown by the enhanced expression of CD80, CD86, and HLADR in LPS‐matured DCs (Figure 1b). No additional induction of maturation was observed in cocultured LPS‐matured DCs after 24 hr. Prolonging the culturing period again did not affect the maturation of immature DCs cultured with chondrogenic hBMSC pellets (Figure S2). There was little difference in the expression of CD80, CD86, and HLADR in immature DCs cultured with chondrogenic hBMSCs over time. By 72‐hr, maturation marker expression in LPS‐matured DCs reduced. Furthermore, the percentage of CD11c positive cells began to diminish after 72 hr in LPS‐matured DCs (Figure S3). These results demonstrate that chondrogenically differentiated hBMSC pellets do not induce DC maturation.

### 3.2 Chondrogenically differentiated hBMSC pellets do not inhibit the antigen uptake capacity in immature DCs

**Figure 2:**
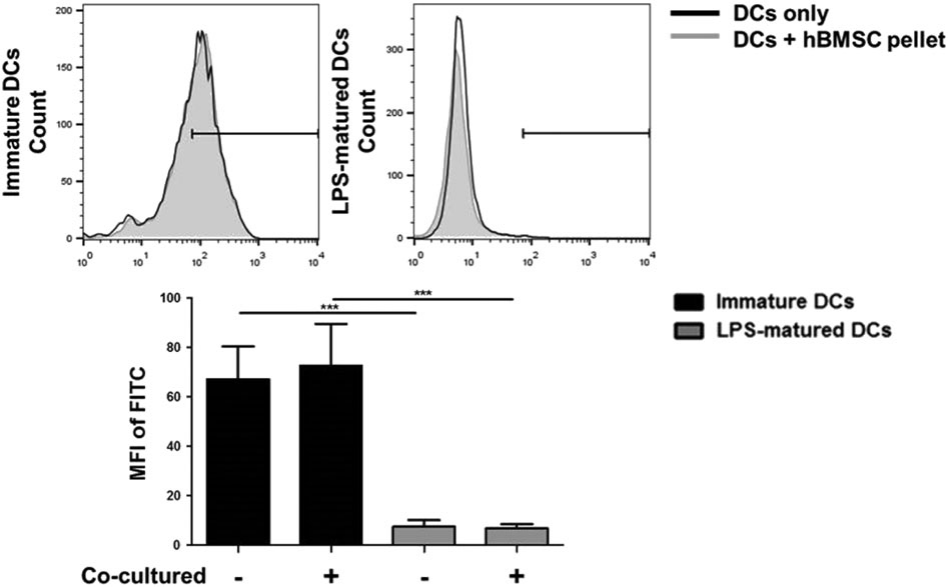
Chondrogenic human bone marrow stromal cell (hBMSC) pellets do not inhibit antigen uptake capacity in immature dendritic cells (DCs). Immature and lipopolysaccharide (LPS)‐matured DCs were harvested following 24 hr coculture with chondrogenically differentiated hBMSC pellets and incubated with fluorescein isothiocyanate (FITC)‐conjugated dextran for 1 hr at 37or 4 °C as a negative control. Chondrogenic hBMSC pellets did not affect antigen uptake in immature DCs. LPS‐matured DCs alone and those cultured with chondrogenic hBMSC pellets demonstrated reduced FITC‐dextran uptake as a result of DC maturation. Chondrogenic hBMSC pellets did not affect FITC‐dextran uptake in LPS‐matured DCs. Data represented as the mean fluorescence intensity (MFI) ± SD. Horizontal bars represent positive regions gated from 4 °C negative controls. *n* = 9 (three different hBMSC and DC donors in triplicate). Linear mixed model with Bonferroni post‐test ^*^ *p* < .05, ^**^ *p* < .005, and ^***^ *p* < .001

Antigen uptake capacity as a functional characteristic of immature DCs was assessed. Upon maturation, DCs lose the capacity to take up antigen. To assess the ability of chondrogenically differentiated hBMSC pellets to effect DC antigen uptake capacity, immature and LPS‐matured DCs cocultured with chondrogenically differentiated hBMSC pellets were incubated for 1 hr at 37 °C with FITC‐conjugated dextran. The uptake of dextran by the DCs was analysed by flow cytometry. No significant increase in dextran uptake was found in immature DCs cultured with chondrogenic hBMSC pellets compared with the DCs alone (Figure 2). This demonstrates that immature DCs have a preserved status regarding taking up antigen and do not mature as a result of the chondrogenic hBMSC pellets. No difference was found in the LPS‐matured DCs cultured with chondrogenic hBMSC pellets. The level in the LPS‐matured DCs (without pellets) was found to be significantly lower than immature DCs, showing successful induction of maturation in these cells (84% decrease in mean fluorescence intensity). Coculturing the cells for longer time again did not affect antigen uptake capacity of both immature and LPS‐matured DCs (Figure S3), indicating that chondrogenically differentiated hBMSC pellets do not affect DC antigen uptake.

### 3.3 Immature CD11c positive DCs surround the periphery of chondrogenic hBMSC pellets

**Figure 3:**
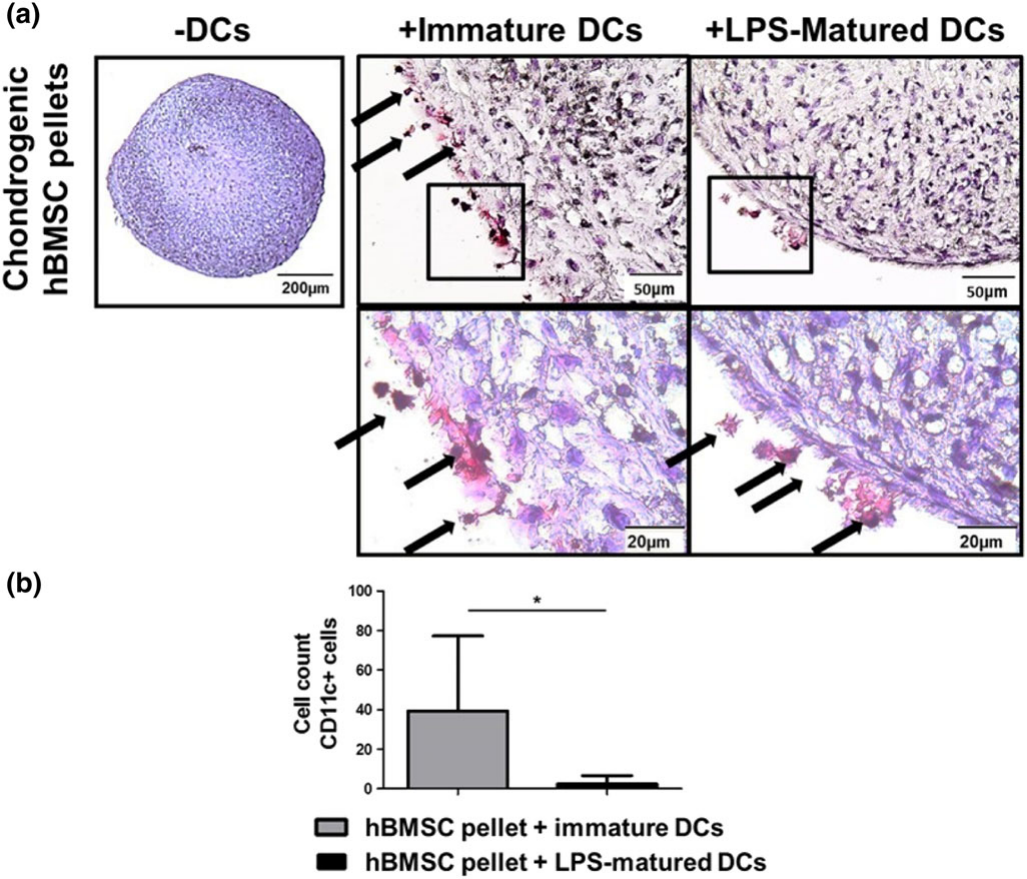
CD11c positive cells were found surrounding the periphery of chondrogenic human bone marrow stromal cell (hBMSC) pellets cultured with immature dendritic cells (DCs). (a) CD11c immunohistochemistry was performed on chondrogenic hBMSC pellets cultured with immature and lipopolysaccharide (LPS)‐matured DCs. (b) The total number of CD11c positive cells (indicated by black arrows) were counted. *n* = 9 (three different hBMSC and DC donors in triplicate) ± SD. Paired *t* test ^*^ *p* < .05, ^**^ *p* < .005, and ^***^ *p* < .001 [Colour figure can be viewed at http://wileyonlinelibrary.com]

To address the characteristic that DCs stay in proximity to chondrogenically differentiated hBMSC pellets, hBMSC pellets cocultured with immature and LPS‐matured DCs were stained for CD11c. CD11c positive cells were found surrounding the periphery of chondrogenic hBMSC pellets cultured with both immature and LPS‐matured DCs (Figure 3a); however, a greater number of positive cells were found on the pellets cultured with immature DCs (Figure 3b). Chondrogenic hBMSC pellets alone were negative for CD11c. This result suggests that immature DCs are interacting with the chondrogenic hBMSC pellets more than LPS‐matured DCs.

### 3.4 Chondrogenic hBMSC pellets do not induce DC migration over time.

**Figure 4:**
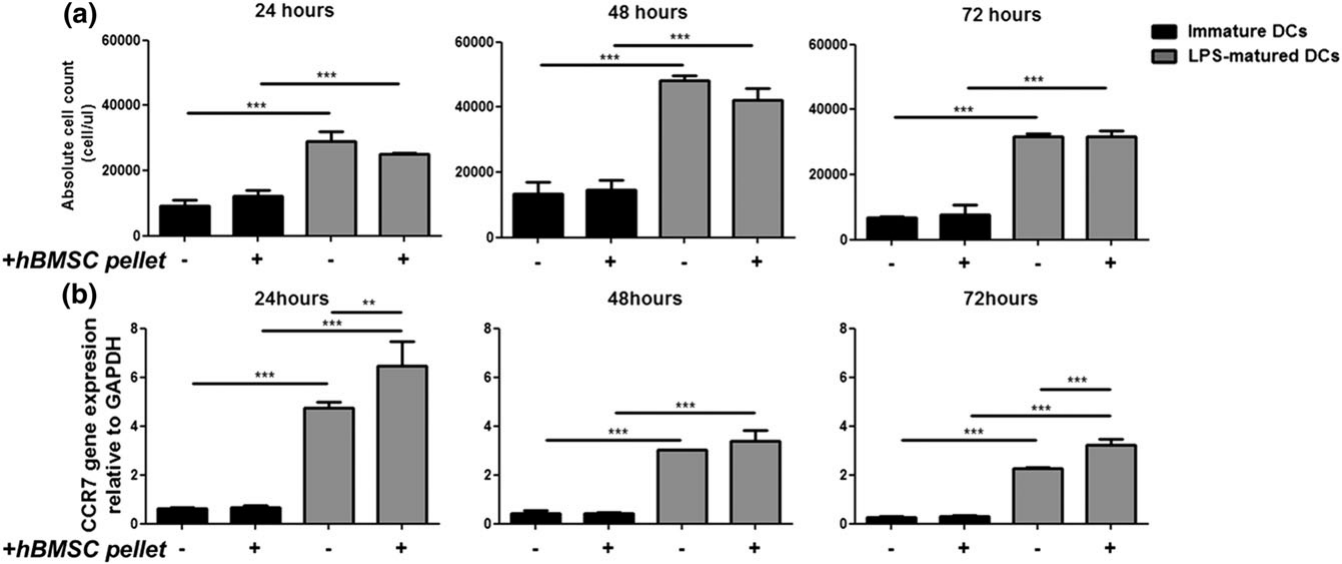
Chondrogenically differentiated human bone marrow stromal cell (hBMSC) does not induce dendritic cell (DC) migration over time. Immature (black) and lipopolysaccharide (LPS)‐matured (grey) DCs were harvested following 24, 48, and 72 hr of coculture with chondrogenically differentiated hBMSC pellets. The DCs from the coculture were seeded in the upper chamber of the transwell system, and CCL21 or control medium was placed in the lower chamber. DCs were incubated for 3 hr at 37 °C. The cells that had migrated were harvested, and an equal amount of counting beads was added to the samples. Samples were analysed by flow cytometry, and the absolute cell count for each sample was calculated. (a) The number of cells spontaneously migrating to control medium was subtracted from the cells that migrated to CCL21. (b) The level of CCR7 gene expression was measured in immature and LPS‐matured DCs cultured with chondrogenically differentiated hBMSC pellets. *n* = 3 (one hBMSC and one DC donor in triplicate at three timepoints) ± SD. Linear mixed model with Bonferroni post‐test ^*^ *p* < .05, ^**^ *p* < .005, and ^***^ *p* < .001

Activated DCs develop migratory capacity. This functionality was tested in a transwell coculture system using immature and LPS‐matured DCs that were cocultured with chondrogenically differentiated hBMSC pellets for 24, 48, and 72 hr. In this system, the responsiveness of both immature and LPS‐matured DCs to CCL21 (a chemokine that attracts mature DCs) was assessed. Immature and LPS‐matured DCs (cultured alone) or those cocultured with chondrogenically differentiated hBMSC pellets were seeded in the upper chamber of the transwell and CCL21, or control medium was placed in the bottom chamber. After 3 hr, the absolute cell number was determined by flow cytometry. After 24 hr in coculture, a slightly higher number of immature DCs migrated in coculture with chondrogenically differentiated hBMSC pellets; however, this was not significant (9,145 ± 1,783 vs. 12,204 ± 1,706 cells; Figure 4a). By 48 and 72 hr, there was still no significant difference in the migration of immature DCs and those cocultured with chondrogenic hBMSCs. In the LPS‐matured DCs, there was a trend towards less migration in coculture with chondrogenic hBMSC pellets at 24 hr (29,046 ± 2,934 vs. 25,104 ± 197 cells) and 48 hr in coculture (48,065 ± 1,600 vs. 42,269 ± 4,604 cells), but this was not statistically significant. No difference in migration was found in LPS‐matured DCs cocultured with chondrogenic hBMSC pellets for 72 hr (31,754 ± 865 vs. 31,792 ± 1,600 cells). Uptake and presentation of antigen of DCs during the immune response also lead to the upregulation of CCR7 (Forster, Davalos‐Misslitz, & Rot, 2008). No difference was found in the CCR7 expression between immature DCs and those cultured with chondrogenic hBMSC pellets (Figure 4b). CCR7 gene expression was upregulated in LPS‐matured DCs cocultured with chondrogenic hBMSC pellets following 24 hr in coculture, and this was maintained for 72 hr. As expected, chondrogenic hBMSC pellets did not express CCR7 from any culture conditions (data not shown). These results show that chondrogenic hBMSC pellets do not induce DC migration.

### 3.5 IL‐6 is secreted at high levels in the supernatants from cocultures of chondrogenic hBMSC pellets with LPS‐matured DCs

**Figure 5:**
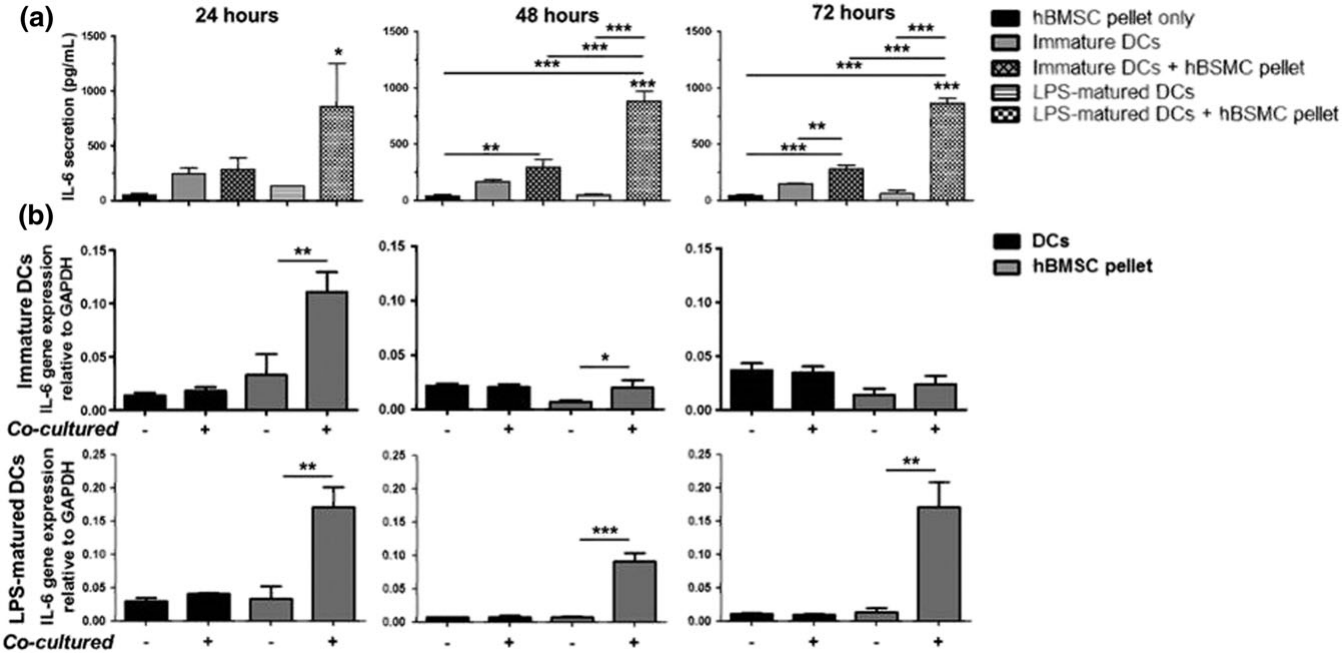
IL‐6 is secreted in supernatants from cocultures of immature and lipopolysaccharide (LPS)‐matured dendritic cells (DCs). (a) Supernatants from immature and LPS‐matured DCs were harvested following 24, 48, and 72 hr in culture with chondrogenically differentiated human bone marrow stromal cell (hBMSCs). *n* = 3 (one different hBMSC and DC donor in triplicate) ± SD. Linear mixed model with Bonferroni post‐test ^*^ *p* < .05, ^**^ *p* < .005, and ^***^ *p* < .001. (c) The level of IL6 gene expression was measured in immature and LPS‐matured DCs cultured alone or with chondrogenically differentiated hBMSC pellets (black) and also in chondrogenic hBMSC pellets cultured alone or with immature and LPS‐matured DCs (grey) over time. Chondrogenic hBMSC pellets expressed higher levels of IL‐6 compared with immature and LPS‐matured DCs. *n* = 3 (one different hBMSC and DC donors in triplicate at three timepoints) ± SD. Unpaired *t* test ^*^ *p* < .05, ^**^ *p* < .005, and ^***^ *p* < .001

DCs produce cytokines upon maturation and activation. Interleukin 6 (IL‐6) is a pleiotropic molecule exhibiting both an anti‐ and pro‐inflammatory effects depending on the situation. Supernatants from the coculture of immature and LPS‐matured DCs cultured with chondrogenically differentiated hBMSC pellets for 24, 48, or 72 hr were harvested. An IL‐6 enzyme‐linked immunosorbent assay was performed on all supernatants. There was no significant difference in the secretion of IL‐6 between immature and LPS‐matured DCs over time. Low levels of IL‐6 were produced from chondrogenically differentiated hBMSC pellets cultured alone. However, in the cocultured conditions, a significantly higher level of IL‐6 production was found in supernatants from cocultures of LPS‐matured DCs and chondrogenically differentiated hBMSC pellets following 24 hr of coculture (Figure 5a). This level of IL‐6 secretion was consistently higher in cocultures of LPS‐matured DCs and chondrogenic hBMSC pellets over time. To investigate which one of the cell types might be responsible for the IL‐6 production, qPCR for IL‐6 was performed on harvested chondrogenic hBMSC pellets and DCs separately. At gene expression level, IL‐6 was found to be upregulated in chondrogenic hBMSC pellets from the coculture of both immature and LPS‐matured DCs after 24 hr (Figure 5b,c). IL‐6 was consistently expressed by chondrogenic hBMSC pellets more than LPS‐matured DCs when cocultured over time. In coculture with immature DCs, chondrogenic hBMSCs expressed higher levels of IL‐6 after 24 hr in coculture, and this level became reduced over time. These results suggest the chondrogenic hBMSCs secrete IL‐6 in response to LPS‐matured DCs.

## 4 DISCUSSION

The immunomodulatory properties of undifferentiated hBMSCs have been widely studied (Aggarwal & Pittenger, 2005; Di Ianni et al., 2008; Di Nicola et al., 2002; Jiang et al., 2005; Nauta, Kruisselbrink, Lurvink, Willemze, & Fibbe, 2006; Roemeling‐van Rhijn et al., 2013; Zhang et al., 2004); however, it is uncertain whether differentiated hBMSCs maintain these immunomodulatory properties. Chondrogenically differentiated hBMSC pellets have been shown to form bone in both immunodeficient and immunocompetent animals (Farrell et al., 2011). The use of allogeneic chondrogenically differentiated hBMSCs could provide a novel “off the shelf” treatment for the repair of bone defects; however, the role of the host cells in the process of the bone formation by allogeneic chondrogenic hBMSCs still needs to be established. Our group has previously shown that chondrogenically differentiated hBMSC pellets do not directly induce T cell responses following 7‐day in vitro culture (Kiernan et al., 2016). Due to the fact that DCs are the key players in stimulating T cell responses, this study investigated the effect of allogeneic chondrogenically differentiated hBMSC pellets on the maturation and function of immature and LPS‐matured DCs in vitro. We have shown that allogeneic chondrogenic hBMSC pellets do not induce DC maturation in vitro.

DCs are known to express the important costimulatory molecules CD80 and CD86 upon maturation, which are ligands for CD28 to induce T cell activation (Sharpe & Freeman, 2002). HLADR (an MHC Class II cell surface receptor) is also expressed to present antigen to T cells via their T cell receptor. Low levels of MHC expression on DCs are known to result in T cell anergy (Chung, Ysebaert, Berneman, & Cools, 2013). To assess the effect of allogeneic chondrogenic hBMSC pellets on DCs, allogeneic chondrogenically differentiated hBMSC pellets were cocultured with immature and LPS‐matured DCs for 24 hr. The levels of CD80, CD86, and HLADR expression in immature DCs cultured with allogeneic chondrogenic hBMSC pellets for 24 hr were not affected. To determine if allogeneic chondrogenic hBMSC pellets would induce maturation over time, the experiment was repeated for 48 and 72 hr. After 48 hr, coculture with chondrogenic hBMSC pellets again did not affect the expression of CD80, CD86, and HLADR in immature DCs or LPS‐matured DCs. Noticeably after 72 hr, the expression of CD11c was diminished in LPS‐matured DCs alone and cultured with allogeneic hBMSC pellets. Furthermore, there were decreases in expression of CD80, CD86, and HLADR in all samples compared with 48 hr. LPS‐matured DCs began to closely resemble the morphology of immature DCs after 72 hr in culture. Long culturing periods of LPS‐matured DCs have been shown to exhaust the cells; however, these cells may still be responsive to other cues and still may have the ability to go on to activate T cells (Abdi, Singh, & Matzinger, 2012; Iwamoto, Ishida, Takahashi, Takeda, & Miyazaki, 2005). LPS has also been shown to induce a population of DCs lowly expressing CD11c that are unable to stimulate T cells but have the capacity to induce regulatory T cells (Wang et al., 2015). We hypothesize that the LPS‐matured DCs cultured alone and with allogeneic chondrogenic hBMSC pellets were maximally stimulated at 48 hr and began to decrease after this time, as seen by the drop in CD11c expression by 72 hr. Further research would be required to determine whether these DCs have the ability to activate T cell responses.

Few studies have studied the effects of chondrogenically differentiated BMSC pellets on DCs. One study investigated the effect of chondrogenically differentiated BMSCs in monolayer on DCs and reported that chondrogenically differentiated BMSCs induce DC maturation shown by increased CD83 expression. Other surface markers such as HLADR, CD14, and CD1a were not significantly different in the cocultures (Chen et al., 2007). Technau, Froelich, Hagen, and Kleinsasser (2011) investigated the immunomodulatory properties of chondrogenically differentiated adipose‐derived stem cells and found upregulation of HLADR and enhanced secretion of the pro‐inflammatory cytokine IFNγ. However, they concluded that chondrogenically differentiated adipose‐derived stem cells have both immunogenic and immunosuppressive properties shown by upregulation of anti‐inflammatory cytokines in the chondrogenic cells. Overall, allogeneic chondrogenically differentiated hBMSC pellets did not affect maturation of DCs.

The effect of allogeneic chondrogenic hBMSCs on the function of immature and LPS‐matured DCs was also assessed. Antigen uptake capacity of DCs has been shown to be inhibited by undifferentiated hBMSCs (Jiang et al., 2005). Chondrogenically differentiated hBMSC pellets again did not induce FITC‐dextran uptake in immature DCs over time. By 72 hr of coculture, there was slightly less FITC‐dextran uptake in immature DCs cocultured with allogeneic chondrogenic hBMSC pellets suggesting that immature DCs were either prevented from taking up antigen or perhaps these immature DCs had become mature by 72‐hr coculture with allogeneic chondrogenic hBMSC pellets. However, the level of FITC‐dextran in immature DCs never reached the levels of LPS‐matured DCs. The level of DC maturity needed for successful activation of T cells would be important to investigate in future studies. Upon maturation, DCs lose the capacity for antigen uptake and are induced to migrate to T cell rich areas of the lymph node. Immature DCs cultured with allogeneic chondrogenic hBMSCs were not induced to migrate compared with the control. Allogeneic chondrogenic hBMSC pellets had no significant effect on LPS‐matured DC antigen uptake or migratory capacity.

Interestingly, it appears from this study that IL‐6 appears to play an important role in the interaction between allogeneic chondrogenic hBMSCs and DCs. IL‐6 is a pleiotropic cytokine known to be essential in the development of APCs (Chomarat, Banchereau, Davoust, & Palucka, 2000; Xing et al., 1998). It has been previously shown that IL‐6 could be responsible for the ability of undifferentiated BMSCs to inhibit DC functions (Djouad et al., 2007; Jiang et al., 2005). Following 24 hr of coculture, it was found that IL‐6 was significantly secreted in supernatants from cultures of LPS‐matured DCs and allogenic chondrogenic hBMSC pellets and this continued over 48 and 72 hr of coculture. At mRNA level, IL‐6 expression was found to be significantly increased by allogeneic chondrogenic hBMSC pellets cultured with LPS‐matured DCs demonstrating that the allogeneic chondrogenic hBMSCs were responsible for the production of the IL‐6. It has been hypothesised that IL‐6 secretion by undifferentiated BMSCs maintains the CD14 monocyte population as opposed to specifically inhibiting the DCs (Jiang et al., 2005). However, all of the DCs were CD14 negative in coculture with chondrogenic hBMSC pellets. Although there was no clear effect of allogeneic chondrogenic hBMSC pellets on maturation or function of LPS‐matured DCs at 24 or 48 hr, IL‐6 production appears to be produced by the pellets in response to LPS‐matured DCs. It is possible that this was why there was no altered behaviour of the DCs. The secretion of IL‐10 and IL‐12 was also measured in the supernatants. IL‐10 was not secreted by either immature DCs or immature DCs cocultured with allogeneic hBMSC pellets after 24 hr (Figure S4). It was secreted by chondrogenically differentiated BMSC pellets cultured alone, LPS‐matured DCs and LPS‐matured DCs cocultured with chondrogenic hBMSC pellets. However, there was no significant difference in the secretion of IL‐10 between cocultures. IL‐12 is known to be involved in the differentiation and function of naïve T cells into effector cells (Hsieh et al., 1993). IL‐12 was found to be secreted in both immature and LPS‐matured DCs cocultured with allogeneic chondrogenically differentiated hBMSCs for 24 hr. However, there was no significant difference in IL‐12 secretion between conditions. Over time, the level of IL‐12 secretion was also measured in supernatants (Figure S5). It was found that IL‐12 was secreted at higher levels in supernatants from cocultured LPS‐matured DCs and chondrogenic hBMSC pellets following 48 and 72 hr, although at a much lower concentration in comparison to IL‐6. The secretion of both IL‐6 and IL‐12 might lead ultimately to the induction of specific T cell responses, or the balance between these cytokines might prevent T cell activation by DCs.

In conclusion, we show for the first time that allogeneic chondrogenic hBMSC pellets do not induce maturation of immature DCs in comparison with LPS‐matured DCs. We did not observe strong immune responses from these DCs in coculture over time. Furthermore, they had no immunomodulatory effects on LPS‐matured DCs; however, IL‐6 appears to be an important cytokine secreted by allogeneic chondrogenic hBMSC pellets in response to LPS‐matured DCs. Hall marks of DC activation are elevated levels of costimulatory molecules, reduced capacity for antigen uptake, migration and the production of cytokines. Here, we show that none of these characteristics were induced in immature DCs cocultured with allogeneic chondrogenic hBMSC pellets. Therefore, complete maturation of immature DCs was not influenced by allogeneic chondrogenic hBMSC pellets. Overall, this study demonstrated that allogeneic chondrogenic hBMSC pellets do not induce DC maturation in vitro and are promising candidates for future bone tissue‐engineering strategies.

## CONFLICT OF INTEREST

The authors have declared that there is no conflict of interest.

## AUTHORSHIP CONTRIBUTIONS

C. H. K., E. F., and P. A. J. B. conceived and designed the study. C. H. K. and E. F. acquired, analysed, and interpreted data and drafted and edited the article. A. K. and M. P. were involved in experimental design, analysis, and interpretation of data. E. B. W. was involved in the conception, design, and critical review of the article. P. A. J. B. was involved in the conception, design, analysis, and interpretation of data and critical review of the article. All authors revised the current article and gave final approval of the current version to be published. All authors are accountable for all aspects of the work in ensuring that questions related to the accuracy and integrity of any part of the work are appropriately investigated and resolved.

## Supporting information


***Supplementary Figure 1*** *– Chondrogenically differentiated hBMSC pellets effect DC maturation over time*. Immature (black) and LPS‐matured DCs (grey) were harvested following 24, 48 and 72 hours of co‐culture with chondrogenically differentiated hBMSC pellets. Flow cytometric analysis illustrated the increased expression of the maturation markers CD80, CD86 and HLADR in immature DCs following 24 hours of co‐culture with chondrogenically differentiated hBMSC pellets (a). The expression of the maturation markers followed a similar trend towards an increase at 48 hours but no difference was observed after 72 hours. The expression of CD80 and 86 increased in LPS‐matured DCs cultured with chondrogenic hBMSCs for 48 and 72 hours (c). Data represented as the mean fluorescence intensity (MFI). Samples were analysed on separate days based on unstained control. *n* = 3 (1 different hBMSC and DC donors in triplicate at 3 timepoints) ± SD Unpaired t‐test *p < 0.05, **p < 0.005, ***p < 0.001
***Supplementary Figure 2** – LPS‐matured DCs lose CD11c expression after 72 hours in culture*. Supplementary figure 3 illustrates the representative plots from the flow cytometry results of the extended culture of immature (a) and LPS‐matured DCs (b). The level of CD11c expression became depleted on LPS‐matured DCs alone and also those co‐cultured with chondrogenic hBMSC pellets following 72 hours in culture.
***Supplementary Figure 3** – Chondrogenically differentiated hBMSC pellets do not continue to induce antigen uptake over time*. Immature (black) and LPS‐matured (grey) DCs were harvested following 24, 48 and 72 hours of co‐culture with chondrogenically differentiated hBMSC pellets and incubated with FITC‐conjugated dextran for 1 hour at 37°C or 4°C as a negative control. Immature DCs cultured with chondrogenic hBMSC pellets did not continue to take up antigen over time compared to immature DCs only (a). There was a reduction in the level of FITC‐dextran uptake in immature DCs cultured with chondrogenically differentiated hBMSC pellets after 72 hours. LPS‐matured DCs alone and those cultured with chondrogenic hBMSC pellets demonstrated reduced FITC‐dextran uptake as a result of DC maturation (b). Chondrogenic hBMSC pellets did not affect FITC‐dextran uptake in LPS‐matured DCs after 48 hours of co‐culture however they slightly induced antigen uptake after 72 hours. Data represented as the mean fluorescence intensity (MFI). *n* = 3 (1 hBMSC donor and 1 DC donor in triplicate ±SD). Linear mixed model with Bonferroni post‐test *p < 0.05, **p < 0.005, ***p < 0.001
***Supplementary Figure 4** – Secretion of IL‐10 and IL‐12 was not significantly affected in supernatants of co‐cultures of LPS‐matured DCs and chondrogenic hBMSC pellets*. Supernatants from immature and LPS‐matured DCs were harvested following 24 hours in culture with chondrogenically differentiated hBMSC pellets. There was no significant difference in the secretion of IL‐10 or IL‐12 between conditions. *n* = 9 (3 hBMSC and 3 different DC donors in triplicate) ± SD. Linear mixed model with Bonferroni post‐test *p < 0.05, **p < 0.005, ***p < 0.001
***Supplementary Figure 5** – IL‐12 continued to be secreted over time in supernatants of co‐cultures of LPS‐matured DCs and chondrogenic hBMSC pellets*. Supernatants from immature and LPS‐matured DCs were harvested following 24, 48 and 72 hours in culture with chondrogenically differentiated hBMSCs. IL‐12 was secreted at higher levels in supernatants harvested from co‐culture of immature DCs and chondrogenic hBMSC pellets for 24 hours. The level of IL‐12 was highest in supernatants of LPS‐matured DCs cultured with chondrogenic hBMSC pellets following 48 and 72 hours. IL‐12 was not secreted by chondrogenic hBMSC pellets only. *n* = 3 (1 hBMSC and 1 DC donor in triplicate) ± SD. Linear mixed model with Bonferroni post‐test *p < 0.05, **p < 0.005, ***p < 0.001
