# Supplementary material for: Allogeneic chondrogenically differentiated human bone marrow stromal cells do not induce dendritic cell maturation
Source: J Tissue Eng Regen Med. 2018 Jun 20;12(6):1530–40. doi: 10.1002/term.2682 (PMC6032931; doi:10.1002/term.2682)

Supplementary Figures:

Supplementary figure 1:

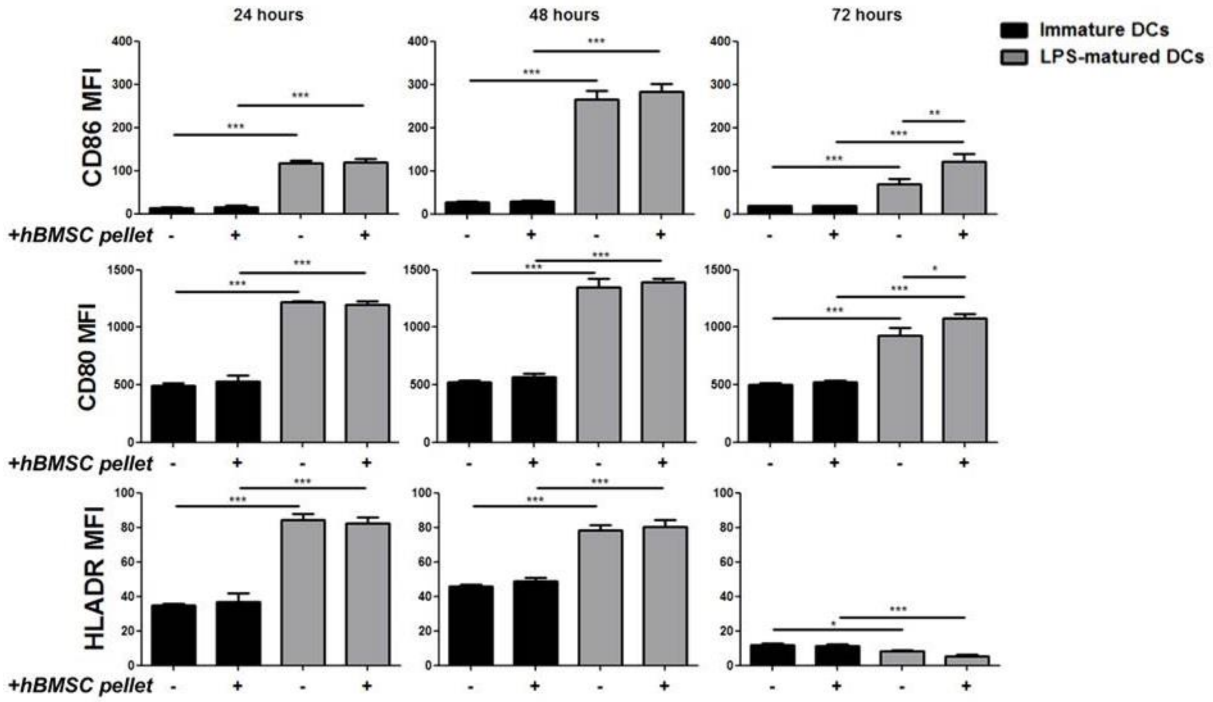

Supplementary figure 2:

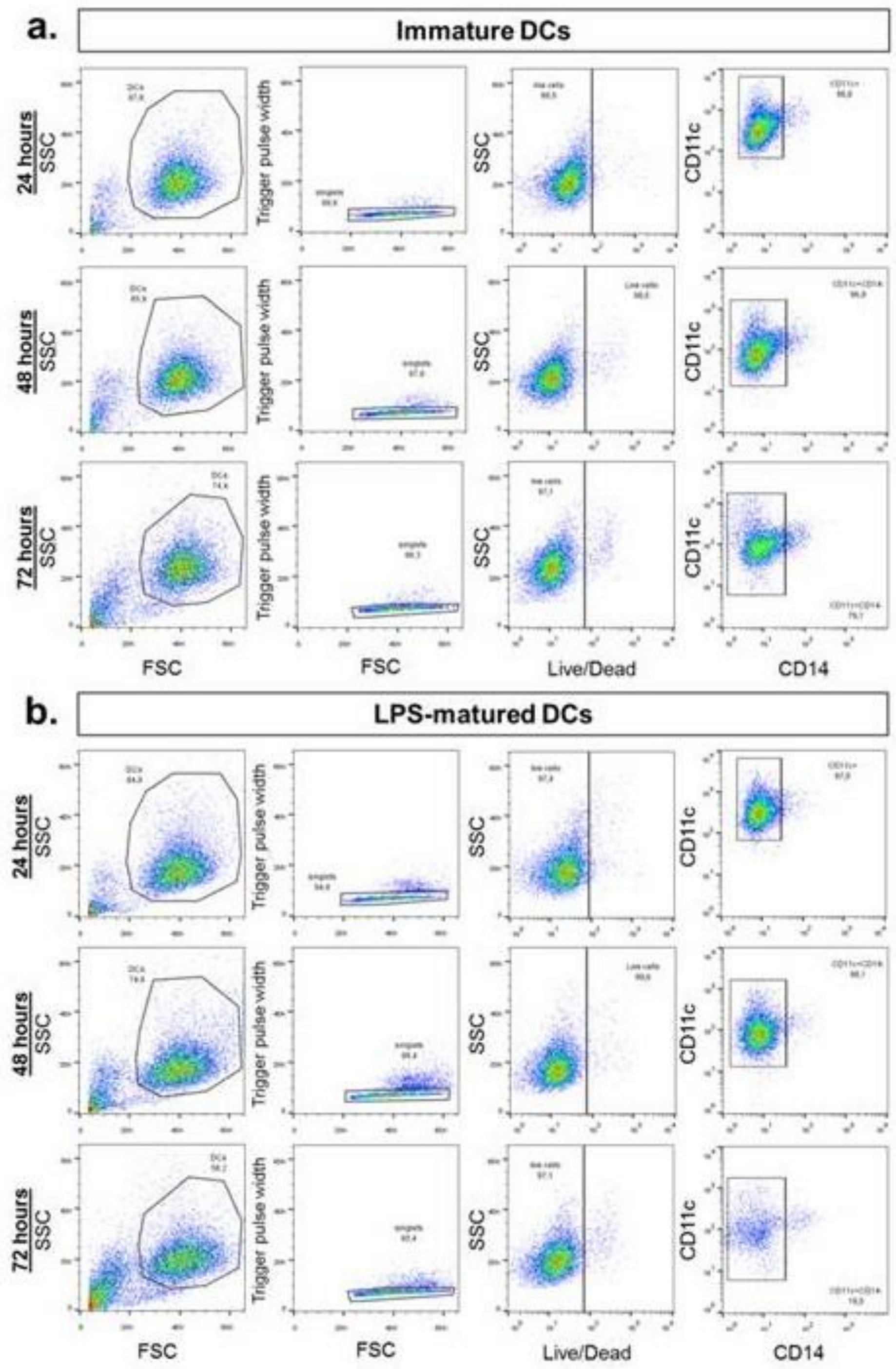

Supplementary figure 3:

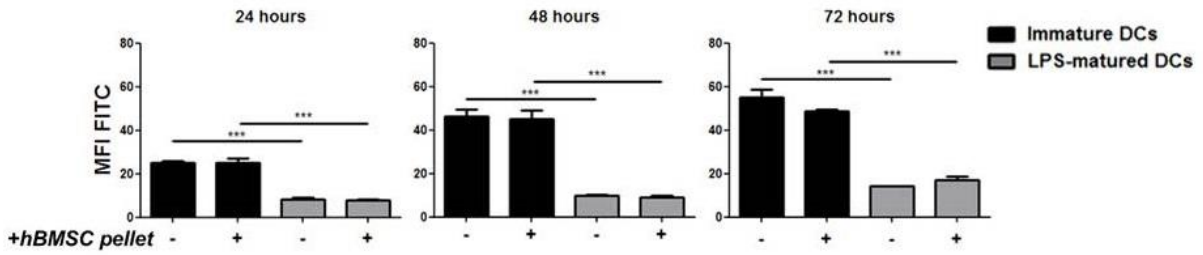

Supplementary figure 4:

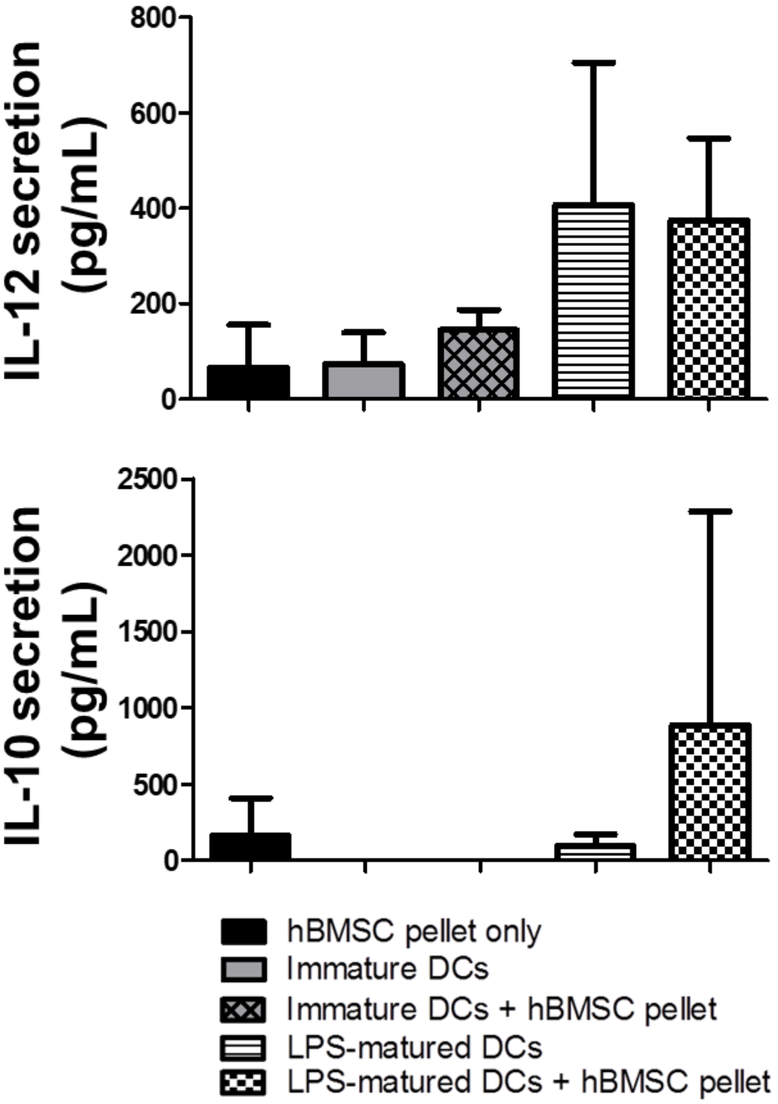

Supplementary figure 5:

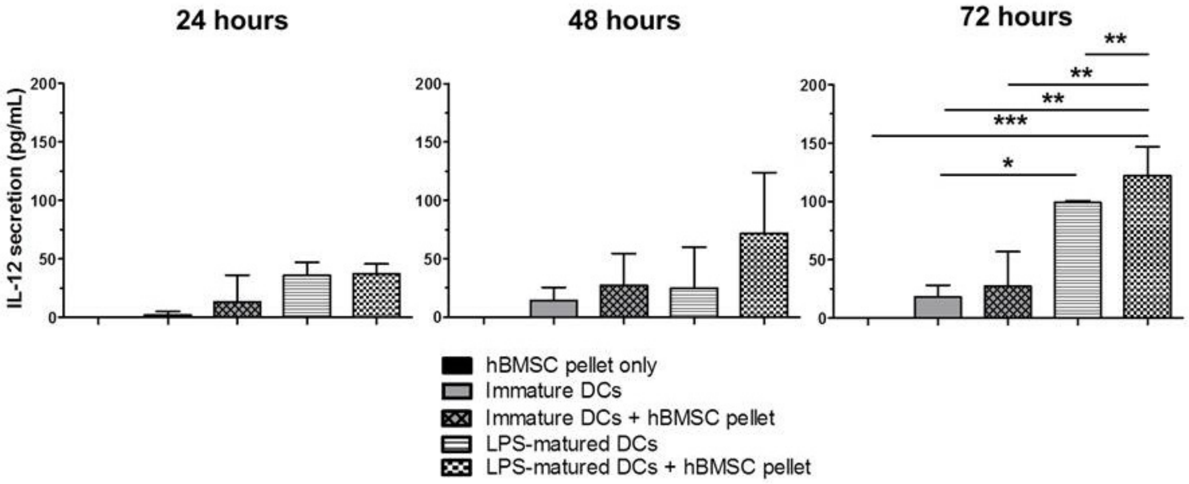

Supplement: Supplementary file 1 — Supplementary Figure 1 – Chondrogenically differentiated hBMSC pellets effect DC maturation over time. Immature (black) and LPS‐matured DCs (grey) were harvested following 24, 48 and 72 hours of co‐culture with chondrogenically differentiated hBMSC pellets. Flow cytometric analysis illustrated the increased expression of the maturation markers CD80, CD86 and HLADR in immature DCs following 24 hours of co‐culture with chondrogenically differentiated hBMSC pellets (a). The expression of the maturation markers followed a similar trend towards an increase at 48 hours but no difference was observed after 72 hours. The expression of CD80 and 86 increased in LPS‐matured DCs cultured with chondrogenic hBMSCs for 48 and 72 hours (c). Data represented as the mean fluorescence intensity (MFI). Samples were analysed on separate days based on unstained control. n = 3 (1 different hBMSC and DC donors in triplicate at 3 timepoints) ± SD Unpaired t‐test *p < 0.05, **p < 0.005, ***p < 0.001 Supplementary Figure 2 – LPS‐matured DCs lose CD11c expression after 72 hours in culture. Supplementary figure 3 illustrates the representative plots from the flow cytometry results of the extended culture of immature (a) and LPS‐matured DCs (b). The level of CD11c expression became depleted on LPS‐matured DCs alone and also those co‐cultured with chondrogenic hBMSC pellets following 72 hours in culture. Supplementary Figure 3 – Chondrogenically differentiated hBMSC pellets do not continue to induce antigen uptake over time. Immature (black) and LPS‐matured (grey) DCs were harvested following 24, 48 and 72 hours of co‐culture with chondrogenically differentiated hBMSC pellets and incubated with FITC‐conjugated dextran for 1 hour at 37°C or 4°C as a negative control. Immature DCs cultured with chondrogenic hBMSC pellets did not continue to take up antigen over time compared to immature DCs only (a). There was a reduction in the level of FITC‐dextran uptake in immature DCs cultured with chondrogeni [file TERM-12-1530-s001.zip › Supplementary Figures.pdf]
